# Occurrence of porcine cysticercosis in free-ranging pigs delivered to slaughter points in Arapai, Soroti district, Uganda

**DOI:** 10.4102/ojvr.v82i1.888

**Published:** 2015-06-11

**Authors:** Gerald Zirintunda, Justine Ekou

**Affiliations:** 1 Department of Animal Production and Management, Busitema University, Uganda; 2 Department of Livestock Health and Entomology, Ministry of Agriculture, Animal Industry and Fisheries, Uganda

## Abstract

Poverty, hunger and the need for production of pigs with meagre or zero inputs have made most farmers release their pigs to range freely, thus creating a pig-human cycle that maintains *Taenia solium,* the pig tapeworm and cause of porcine cysticercosis, in the ecosystem. A preliminary study was designed to establish the prevalence of porcine cysticercosis by postmortem examination of the tongue and carcass of free-range pigs from February to April 2014 in Arapai subcounty, Soroti district, eastern Uganda. The tongue of each pig was extended and examined before deep incisions were made and the cut surfaces were examined. The rest of the carcasses were examined for cysts. Out of 178 pigs examined, 32 were qualitatively positive for porcine cysticercosis, representing a prevalence of 18.0%. This high prevalence represents a marked risk to the communities in the study area of neurocysticercosis, a debilitating parasitic zoonosis. Proper human waste disposal by use of pit latrines, confinement of free-range pigs and treatment with albendazole and oxfendazole are recommended.

## Introduction

Pig production has increasingly become an important activity in Uganda, with the pig population increasing in the last three decades from 0.19 million to 3.6 million (Ministry of Agriculture, Animal Industry and Fisheries [MAAIF] & Uganda Bureau of Statistics 2009; Uganda Bureau of Statistics 2013). In comparison to other animal rearing enterprises, pig production requires minimal inputs and relatively smaller space (Eusebio 1980), which makes pig farming popular. It is thus not surprising that more than 1.1 million families, about 18% of the total households in Uganda, own pigs (MAAIF & Uganda Bureau of Statistics 2009).

This rapid increase in production has been matched by a rapid increase in consumption of pork within the country, driven not only by population growth but also by a combination of rising income and changing preferences associated with urbanisation. Uganda has the highest per capita consumption of pork in sub-Saharan Africa, with a 2011 estimate of 3.4 kg/person/year representing a ten-fold increase in the last 30 years (Ballantyne 2012). However, programmes promoting pig production have not emphasised proper management and public health concerns. Poverty and lack of resources have driven farmers or communities to rear pigs extensively with very minimal inputs, hence exposing them to the risk of porcine cysticercosis. Porcine cysticercosis is caused by the metacestodes (cysticerci) of the cestode *Taenia solium,* and it is endemic in Uganda (World Organization for Animal Health 2014; Waiswa *et al.* 2009).

*Taenia solium* is a zonootic tapeworm that is maintained by a pig-human cycle in the ecosystem. The infection is contracted by pigs when they either ingest human faeces containing infective eggs or when feeding on pasture contaminated with *T. solium* eggs (Carrique-Mas *et al.* 2001). If people consume raw or inadequately cooked pork from infected animals, the larval cysts can develop into the adult stage tapeworm in their intestines, where gravid proglottids containing infective eggs detach from the adult tapeworm and are excreted in the faeces (Garcia *et al.* 2003). In places where open defaecation is common the faeces containing these infective eggs are consumed directly or from contaminated pasture by pigs, and the lifecycle is perpetuated (Ito *et al.* 2006; Lescano *et al.* 2007).

Humans can also act as an aberrant intermediate host for *T. solium* if there is faecal-oral contamination with the infective eggs. In such cases the larval stage can be found in human muscle, heart, eyes, skin or central nervous system, causing human cysticercosis (Flisser, Rodríguez-Canul & Willingham 2006). The most serious form of human cysticercosis is when the larval form develops in the brain, a condition called neurocysticercosis (NCC). Adult *T. solium* infestation in humans is associated with subclinical conditions of malnutrition and anasarca due to larval migration through the tissues (Delgado-Azanero *et al.* 2007). Human NCC may manifest with headaches, blindness, hydrocephalus, chronic meningitis and dementia (Carabin *et al.* 2005). NCC contributes to epilepsy in regions where pigs are free-ranging and hygiene is poor (Blocher *et al.* 2011; Rottbeck *et al.* 2013). The prevalence of cysticercosis was determined to be 11.7% amongst patients with epilepsy and 2.8% amongst controls who were normal individuals in families of Burundi (Newell *et al.* 1997), indicating that cysticercosis causes epilepsy.

There is evidence of a high prevalence of NCC infecting people in villages where pigs are raised (Phiri *et al.* 2003). Age increases the risk of being positive for cysticercosis in pigs where open-air defaecation and free-range pig raising are practised (Jayashi *et al.* 2012). Approximately 50 000 individuals die globally every year of NCC caused by the parasitic intermediate stages of *T. solium* (Ito *et al.* 2006). The purpose of the present study was therefore to estimate the prevalence of porcine cysticercosis amongst pigs delivered for slaughter in Arapai in the Soroti district of eastern Uganda.

## Materials and methods

**FIGURE 1:**
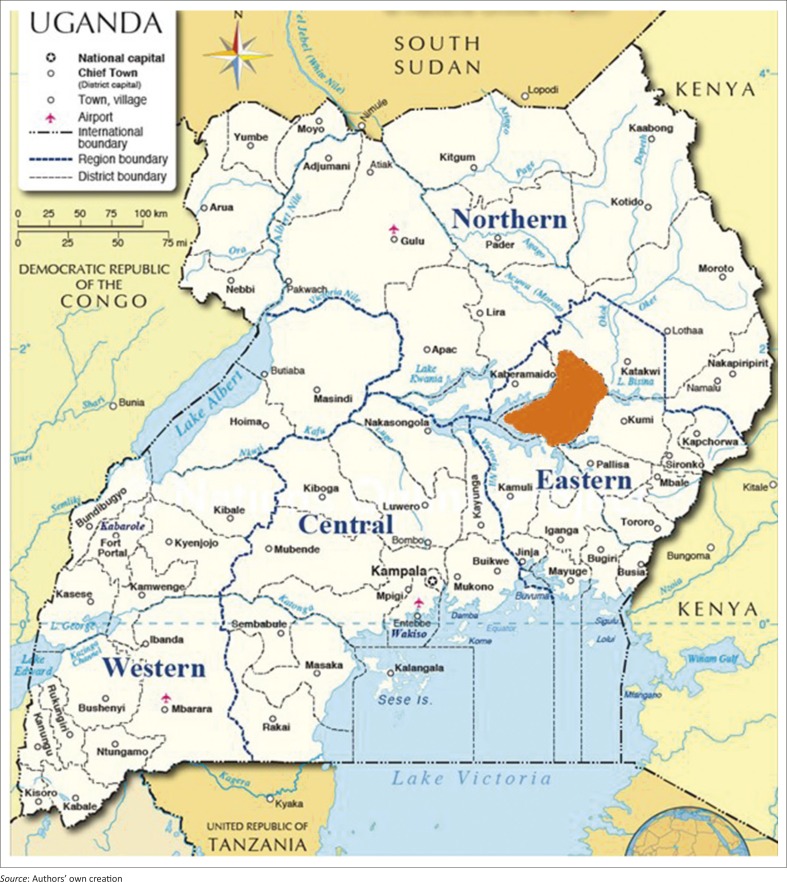
Map of Uganda showing location of Soroti District (shaded red).

**FIGURE 2:**
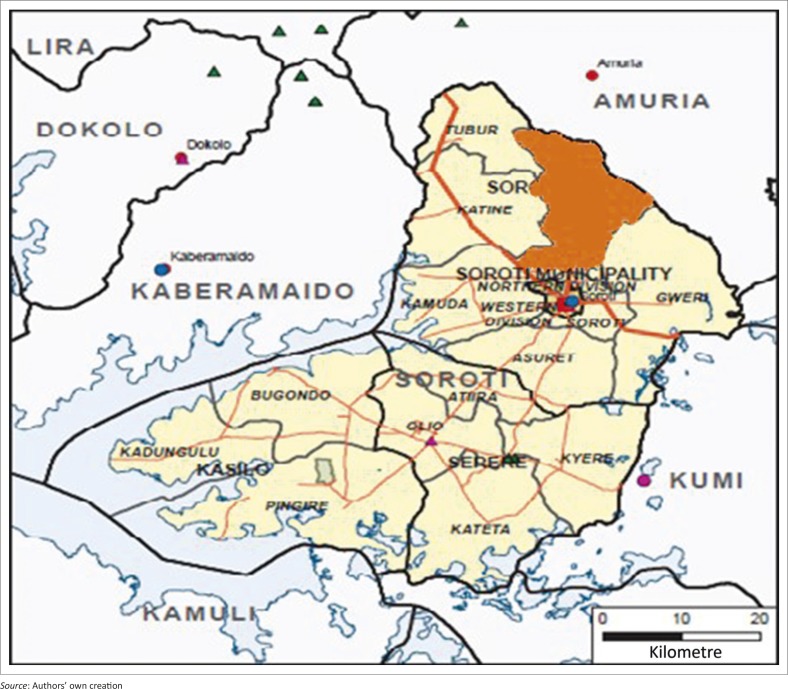
Map Soroti District showing location of Arapai (shaded).

The study was done in Arapai subcounty which is located in the northern part of Soroti district in eastern Uganda. Figures 1 and 2 show the location of Soroti district in Uganda and the location of Arapai subcounty in Soroti.

The 16 most popular slaughter points for pig trade in the subcounty were selected for inclusion in the study, namely five slaughter places in Aloet parish, eight in Arapai market, two in Temele trading centre and one in Apida trading centre. A cross-sectional qualitative study in which the tongue, cardiac muscles and thigh muscles were examined for the presence of cysts was performed. The head of a slaughtered pig was set aside; the tongue was fully extended, dried with a smooth towel and examined for cysts on both the dorsum and ventrum before it was incised and the cut surfaces were examined. Visual inspection of the carcass, its cut surfaces and the organs within it was done. The external and internal masseters and the pterygoid muscles were examined and two incisions made into each, the cuts being parallel to the bone and right through the muscle. The pericardium was examined visually. The heart was incised once lengthwise through the left ventricle and interventricular septum to expose the interior and cut surfaces for examination. In addition two deep incisions were made into the left ventricle. After removal of the peritoneum the muscles of the diaphragm were examined visually and incised once. The oesophagus was examined visually. The gracilis muscle was incised once parallel to the pubic symphisis.

All pigs were examined consistently. Cysts were identified as oval, about 10 mm × 5 mm or larger, with a delicate, fairly translucent, white parasite membrane and host capsule. Within the cyst a pale fluid and the scolex, visible as a white dot within the cyst, usually invaginating midway along the long axis of the cyst, was considered diagnostic (World Organization for Animal Health 2014).

## Results

As shown on Table 1, a total of 178 pigs were examined and 32 were found to be positive for cysticercosis, indicating an overall prevalence of 18.0% in the study area. Aloet and Arapai market recorded similar results of 14.9% and 14.6% respectively, whilst Temele and Apida also had similar results of 28.6% each.

**TABLE 1:** Occurrence of porcine cysticercosis amongst free-range pigs delivered to various slaughter places in Arapai subcounty, Soroti district, eastern Uganda.

Place of slaughter	Number of pigs sampled	Positive cases	% prevalence
Aloet (5 points)	47	07	14.9
Arapai market (8 points)	89	13	14.6
Temele (2 points)	07	02	28.6
Apida centre (1 point)	35	10	28.6
Total	178	32	18.0

## Discussion

The overall observed porcine cysticercosis prevalence in this study was 18.0%. This was higher than the prevalence determined by tongue and necropsy examination previously reported in eastern and southern provinces of Zambia (Mwape *et al.* 2012; Sikasunge *et al.* 2007), the Teso district of Western Kenya (Mutua *et al.* 2007), North West Cameroon (Shey-Njila *et al.* 2003), Angónia district of Mozambique (Pondja *et al.* 2010), Homa Bay district of Kenya (Eshitera *et al.* 2012) and the Eastern Cape Province, South Africa (Krecek *et al.* 2008).

A prevalence as high as 18.0% based on tests of low sensitivity should cause concern. The tongue test is 70% sensitive and 100% specific; the enzyme-linked immunosorbent assay (ELISA) has a sensitivity and specificity of 75%, whilst enzyme-linked immune-electro transfer blot (EITB) has a sensitivity and specificity of 100% (Gonzalez *et al.* 1990). The prevalence may be projected to be about 40% had more sensitive tests such as EITB been used.

Shey-Njila *et al.* (2003) found 4.44% by tongue examination and 27.7% prevalence by ELISA in the same population. Sikasunge *et al.* (2007) found 12.7% by tongue examination and 32.1% by ELISA, Pondja *et al.* (2010) 12.7% by tongue examination and 34.9% by ELISA, Eshitera *et al.* (2012) 5.6% by tongue examination and 32.6% by ELISA, Krecek *et al.* (2008) 11.9% by tongue examination and 33.3% by EITB, whilst Gonzalez *et al.* (1990) found 23.4% by tongue examination, 37.7% by ELISA and 51.9% by EITB.

Thus the observed prevalence was very high given the low sensitivity of the test methods employed, which is seriously concerning since the true prevalence is probably much higher.

The higher prevalence in Arapai subcounty was thought to be due to the very low latrine coverage and the free-ranging rearing system, which enables pigs to scavenge in the environment and to consume human faeces. In Soroti pit latrine coverage is 71% and 94% of pigs are reared either free-ranging or tethered in bushes where they are at risk of acquiring porcine cysticercosis (Uganda Bureau of Statistics 2009; Zirintunda 2011). Most farmers are poor and food insecure, and equally their pigs lack sufficient food, as reported in other studies (Adesehinwa, Makinde & Oladele 2003; Chimonyo *et al.* 2005; Halimani *et al.* 2012). The free-ranging pigs are also able to move long distances away from their owners’ premises where they access eggs of *T. solium,* even if their owners are free from infection and have access to latrines.

The pigs are not fed commercial rations since most owners also have very little to eat, and therefore pigs are exposed to human faeces whilst scavenging. The farmers in Soroti have no regular strategies to control worms amongst themselves or amongst their pigs. This could lead to a higher prevalence of cysticercosis when compared to other places where deworming programmes have been implemented.

The prevalence of cysticercosis was similar for Aloet and Arapai sub-counties and for Temele and Apida slaughter points; this was possibly because of almost the same magnitude of those factors that affect porcine cysticercosis, like latrine coverage and use. Aloet and Arapai slaughter points had a slightly lower prevalence compared to Temele and Apida. Aloet is a township with moderate pit latrine coverage whilst Arapai is a cattle market with some sanitary facilities. In addition, both places receive pigs for slaughter from distant places and through middlemen who probably carry out pre-transit lingual examination of the pigs before delivery (Nsadha *et al.* 2014).

## Conclusion

In conclusion, the prevalence of porcine cysticercosis in Arapai, Soroti district is very high. Since it is a zonoosis, the human population in Arapai is at a high risk of the maladies associated with porcine cysticercosis, like NCC and epilepsy. It is also possible for cysticercosis to occur in people without brain involvement, and for clinical symptoms to appear to be absent (Somers *et al.* 2006). Confinement of pigs should be adopted to prevent continuous transmission of porcine cysticercosis (Pouedet *et al.* 2002). Vaccination of pigs with crude extracts of *T. solium* metacestodes and oncosphere antigens (Flisser *et al.* 2004; Molinari *et al.* 1997) may also be helpful. Treatment with drugs such as albendazole and oxfendazole is of value, as the cysts may lose their fluid and collapse.
